# Cytotoxic Compounds Isolated from *Murraya tetramera* Huang

**DOI:** 10.3390/molecules190913225

**Published:** 2014-08-27

**Authors:** Chun-Xue You, Kai Yang, Cheng-Fang Wang, Wen-Juan Zhang, Ying Wang, Jiao Han, Li Fan, Shu-Shan Du, Zhu-Feng Geng, Zhi-Wei Deng

**Affiliations:** 1 State Key Laboratory of Earth Surface Processes and Resource Ecology, Beijing Normal University, Haidian District, Beijing 100875, China; E-Mails: youchunxue@mail.bnu.edu.cn (C.-X.Y.); yangk_1988@mail.bnu.edu.cn (K.Y.); wangchengfang@mail.bnu.edu.cn (C.-F.W.); zwj0729@mail.bnu.edu.cn (W.-J.Z.); yingw150@163.com (Y.W.); 2 State Key Laboratory Breeding Base of Dao-di Herbs, China Academy of Chinese Medical Sciences, Dongcheng District, Beijing 100700, China; 3 China CDC Key Laboratory of Radiological Protection and Nuclear Emergency, National Institute for Radiological Protection, Chinese Center for Disease Control and Prevention, Xicheng District, Beijing 100088, China; 4 Analytical and Testing Center, Beijing Normal University, Haidian District, Beijing 100875, China; E-Mails: hanjiao19890516@163.com (J.H.); gengzhufeng@bnu.edu.cn (Z.-F.G.); dengzw@bnu.edu.cn (Z.-W.D.)

**Keywords:** *M. tetramera*, cytotoxicity, coumarin, sesquiterpene

## Abstract

A new compound and seven known compounds were isolated from *Murraya tetramera* Huang for the first time, and they were identified with NMR and MS spectral analysis. It was confirmed that the new compound was 10-methoxy-7-methyl-*2H*-benzo[g]chromen-2-one (**3**) and the others were *β*-eudesmol (**1**), *trans*-3*β*-(1-hydroxy-1-methylethyl)-8*aβ*-methyl-5-methylenedecalin-2-one (**2**), 5,7-dimethoxy-8-[(*Z*)-3'-methyl-butan-1',3'-dienyl]coumarin (**4**), 7-geranyloxy-6-methoxycoumarin (**5**), 5,7-dimethoxy-8-(3-methyl-2-oxo-butyl)coumarin (**6**), murrangatin acetate (**7**) and toddalenone (**8**). Furthermore, the cytotoxic activity against human lung adenocarcinoma (A549), human hepatocellular carcinoma cells (SMMC-7721), human bladder tumor cells (EJ), human cervical carcinoma cells (HeLa), and human B-lineage acute lymphoblastic leukemia 1 cells (BALL-1) was evaluated for all compounds. It was found that five of them displayed various degrees of cytotoxicity against different testing targets. Compound **1** showed significant cytotoxic activity against the five cell lines (A549, SMMC-7721, EJ, Hela and BALL-1). Compounds **2** and **5** showed significant cytotoxicity against three cell lines (A549, SMMC-7721 and BALL-1). Compound **4** showed significant cytotoxicity against three cell lines (A549, EJ and BALL-1). However, compound **3** only showed fair cytotoxicity against the BALL-1 cell line. The structure-active relationships were investigated as well. These active compounds might be potential lead compounds for the treatment of cancer.

## 1. Introduction

Cancer is one of the most common diseases that threaten peoples’ health. Accordingly, much effort has been invested to develop effective treatments. Chemotherapy and radiotherapy have been the primary approaches for conventional cancer treatment, but they are not always effective [1,2,3]. Traditional Chinese medicines (TCMs) are generally economical and plentiful, while showing very low toxicity or side effects in clinical practice. Hence, TCMs have been applied worldwide for the treatment of cancers [3,4,5,6]. Furthermore, TCM have been one of the most important sources for seeking new leading compounds that possess significant cytotoxicity [7,8,9].

In East Asia, the genus *Murraya* (family Rutaceae) has been widely used in traditional medicine. Its plants contain various alkaloids, coumarins and flavonoids [10,11]. The cytotoxicity has been investigated in some species in this genus, such as *M. koenigii* [12], *M. paniculata* [13,14], *M. euchrestifolia* [15] and *M. exotica* [16]. However, there are no reports on the cytotoxicity of *M. tetramera* Huang.

*M. tetramera* is widely distributed in the Chinese provinces of Guangxi and Yunnan. It has been used as a folk TCM for the treatment of colds, coughs, asthma, stomach disorders, rheumatism, pruritus and eczema [11,17]. In this work, we sought to isolate and identify bioactive compounds with potential cytotoxicity from *M. tetramera*. As a result a new coumarin and seven known compounds were isolated from *M. tetramera* for the first time and all the compounds were evaluated *in vitro* against the A549, SMMC-7721, EJ, HeLa and BALL-1 tumor cell lines. The results indicated that some of these compounds have significant cytotoxic activities against the five tested human cancer cell lines.

## 2. Results and Discussion

### 2.1. Compounds Isolated from M. tetramera

**Figure 1:**
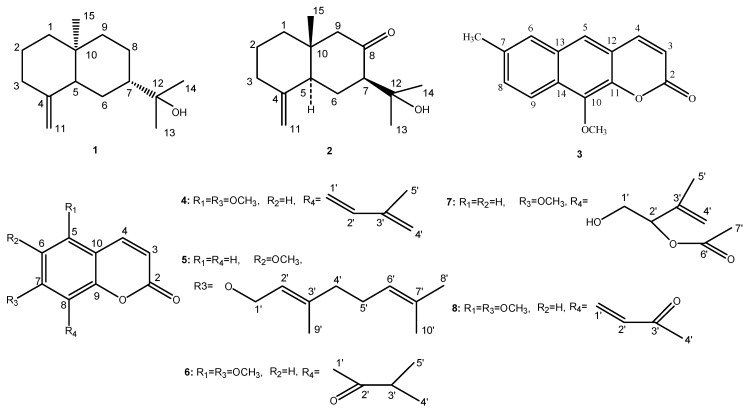
The structures of compounds **1**–**8**.

A new compound and seven known compounds were isolated from the *M. tetramera* for the first time. The new one was identified that 10-methoxy-7-methyl-*2H*-benzo[g]chromen-2-one (**3**) and the others were *β*-eudesmol (**1**), *trans*-3*β*-(1-hydroxy-1-methylethyl)-8*aβ*-methyl-5-methylenedecalin-2-one (**2**), 5,7-dimethoxy-8-[(*Z*)-3'-methylbutan-1',3'-dienyl]coumarin (**4**), 7-geranyloxy-6-methoxycoumarin (**5**), 5,7-Dimethoxy-8-(3-methyl-2-oxo-butyl)coumarin (**6**), murrangatin acetate (**7**) and toddalenone (**8**). Compounds **1** and **2** are sesquiterpenes and compounds **3**–**8** are coumarins. Their structures are shown in Figure 1.

### 2.2. Chemical Structure Identification of the New Compound

**Figure 2:**
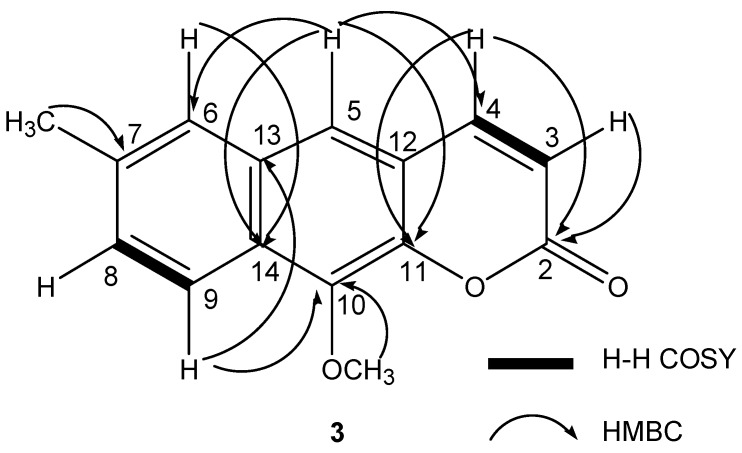
The structure of compound **3** and key assignments of its COSY and HMBC correlations signals.

Compound **3** was obtained as colorless needles. The molecular formula was established as C_15_H_12_O_3_ by HR-ESI-MS, which indicted an [M+H]^+^ peak at *m*/*z* 241.0861 (calculated for C_15_H_13_O_3_, 241.0865). The ^1^H-NMR spectrum showed characteristic peaks of a coumarin framework at δ_H_ 8.21 (1H, d, *J* = 9.5 Hz, H-4), δ_H_ 6.86 (1H, s, H-5), δ_H_ 6.47 (1H, d, *J* = 9.5 Hz, H-3) indicative of a substituent at C-13, C-14 and C-10. Moreover, the ^1^H-NMR spectrum showed one methoxyl peak at δ_H_ 4.03 (3H, s, 10-OCH_3_) and one aromatic methyl peak at δ_H_ 2.54 (3H, s, 7-CH_3_). The ^13^C-NMR spectrum revealed the presence of fifteen carbon atoms and the characteristic coumarin framework ones at δ_C_ 161.03 (C-2) and δ_C_ 152.46 (C-11). The H-H COSY spectrum exhibited the correlations between H-3 (δ_H_ 6.47) and H-4 (δ_H_ 8.21), between H-8 (δ_H_ 7.32) and H-9 (δ_H_ 8.33). The HMBC spectrum showed correlations arising from H-3 (δ_H_ 6.47) to C-2 (δ_C_ 161.0), H-4 (δ_H_ 8.21) to C-2 (δ_C_ 161.0) and C-11 (δ_C_ 152.5), H-5 (δ_H_ 6.86) to C-6 (δ_C_ 126.0), C-4 (δ_C_ 139.4), C-14 (δ_C_ 117.1) and C-11 (δ_C_ 152.5), H-6 (δ_H_ 7.53) to C-14 (δ_C_ 117.1), 7-CH_3_ (δ_H_ 2.5) to C-7 (δ_C_ 126.9), H-9 (δ_H_ 8.33) to C-10 (δ_C_ 152.6) and C-13 (δ_C_ 135.8), 10-OCH_3_ (δ_H_ 4.03) to C-10 (δ_C_ 152.6). The H–H COSY and HMBC correlations were presented in Figure 2. On the basis of the results, the structure of compound **3** was identified as 10-methoxy-7-methyl-*2H*-benzo[g]chromen-2-one.

### 2.3. Cytotoxic Activity of Isolated Compounds

The cytotoxicity of compounds **1**–**8** was evaluated against A549, SMMC-7721, EJ, HeLa and BALL-1 cancer cell lines using the Cell Counting Kit (CCK)-8 method. The results are listed in Table 1. The two sesquiterpenes showed fair cytotoxicity against the five cell lines. Moreover, compound **1** possessed stronger cytotoxic activity against A549, SMMC-7721, EJ, HeLa and BALL-1, with IC_50_ values of 6.70, 5.17, 31.93, 17.82 and 11.15 µg/mL, respectively. Coumarins **3**–**8** share the same basic skeleton with different substitution patterns, yet their cytotoxic activities varied greatly. Compound **5** exhibited potent cytotoxicity against A549, SMMC-7721 and BALL-1, with IC_50_ values of 7.30, 9.09 and 12.50 µg/mL. Compound **4** exhibited potent cytotoxicity against A549, EJ and BALL-1 with IC_50_ values of 17.04, 30.59 and 22.54 µg/mL, respectively. Compound **3** merely exhibited cytotoxic activity against BALL-1 with an IC_50_ value of 94.88 µg/mL. None of the tested cell lines were susceptible to compounds **6**–**8**.

**Table 1:** Cytotoxicity of compounds **1**–**8** from *Murraya tetramera*.

Compound	IC_50_ (µg/mL) ^a^				
	A549	SMMC-7721	EJ	Hela	BALL-1
**1**	6.70 ± 1.05	5.17 ± 0.97	31.93 ± 2.84	17.82 ± 2.34	11.15 ± 1.62
**2**	31.67 ± 2.36	35.62 ± 2.73	47.45 ± 3.22	70.61 ± 3.95	33.91 ± 2.78
**3**	>100	>100	>100	>100	94.88 ± 3.25
**4**	17.04 ± 0.58	>100	30.59 ± 2.73	>100	22.54 ± 2.03
**5**	7.30 ± 0.46	9.09 ± 0.51	38.18 ± 2.23	46.63 ± 2.62	12.50 ± 1.47
**6**–**8**	>100	>100	>100	>100	>100
**DOX ^b^**	3.53 ± 0.25	1.35 ± 0.28	5.88 ± 0.18	2.11 ± 0.21	6.99 ± 0.37

^a^ Inhibitory activity was expressed as the mean ± SD of 50% inhibitory concentration of triplicate determinations and was obtained by interpolation of concentration-inhibition curve. ^b^ Doxorubicin (positive control).

The various cytotoxic activities might be related to the different substitution patterns in the chemical structures. Among compounds **3**–**8**, compound **5** had a longer alkyl-substituent than the other compounds and it showed the most potent cytotoxic activity against the human cancer cell lines, which corresponds to the result previously described by Wang *et al.* [18] indicating that the length of alkyl-substituents contributed to the cytotoxicity. Interestingly, the results also showed that the compounds possessing carbonyls on the alkyl moiety had weak cytotoxic activities. Further study is needed to investigate the structure-active relationships.

## 3. Experimental Section

### 3.1. General Information

^1^H- and ^13^C-NMR and 2D-NMR spectra were recorded on Bruker Avance III NMR spectrometer with the magnetic field of 11.74 Tesla. HR-ESI-MS were obtained on a Bruker Q-TOF mass spectrometer. Silica gel (160–200 mesh) used for column chromatography and TLC (silica gel G plates) used for monitoring fractions were purchased from Qingdao Marine Chemical Plant (Qingdao, China). Sephadex LH-20 was supplied by Amersham Pharmacia Biotech (Beijing, China). Analytical grade solvents were produced by Beijing Chemical Factory (Beijing, China).

### 3.2. Plant Material

The branches with leaves of *M. tetramera* were collected in June 2012 from Xishuangbanna, Yunnan Province, China (21.13°~22.60° N latitude, 99.93°~101.83° E longitude). The plant was identified by Dr. Liu, Q.R. (College of Life Sciences, Beijing Normal University, Beijing, China) and a voucher specimen (BNU-CMH-Dushushan-2012-06-017-007) was deposited at the Herbarium (BNU) of College of Resources Sciences, Beijing Normal University.

### 3.3. Extraction and Isolation

The dried samples (2.5 kg) were extracted with petroleum ether-ethyl acetate (PE/EtOAc, 20 L) three times (each for half an hour) under ultrasound. A crude extract (100.62 g) was obtained by solvent evaporation under vacuum. The extract was fractionated by silica gel column chromatography (160–200 mesh, 10.0 × 33 cm, 1000 g), using a gradient solvent system of PE/EtOAc (100:1, 80:1, 60:1, 40:1, 20:1, 10:1, 5:1, 1:1 and EtOAc) to afford 90 fractions. Fractions with similar TLC patterns were combined. 160–200 Mesh/Fr. 29–30 (1.55 g) and 160–200 mesh/Fr. 35–37 (1.41 g) were chromatographed on a silica gel column eluting with PE/EtOAc (60:1) to obtain compound **1** (128.3 mg) and compound **3** (16.7 mg), respectively. 160–200 Mesh/Fr. 51 (0.88 g) and 160–200 mesh/Fr. 54–57 (1.17 g) were subjected to repeated silica gel column chromatography eluting with PE/EtOAC (10:1) to afford compound **2** (11.7 mg) and compound **4** (52.6 mg), respectively. 160–200 Mesh/Fr. 64 (0.41 g) and 160–200 mesh/Fr. 67–70 (3.12 g) were repeatedly subjected to silica gel column chromatography eluting with PE/EtOAc 5:1, and then purified by chromatography on a Sephadex LH-20 column with MeOH as eluent to give compound **5** (17.2 mg) and **6** (62.8 mg), respectively. Compounds **7** (33.7 mg) and **8** (27.9 mg) were obtained from 160–200 mesh/Fr. 74 (3.35 g) and 160–200 mesh/Fr. 77–78 (2.55 g) after repeatedly purification by chromatography on a silica gel column eluting with PE/EtOAc 2:1.

### 3.4. Characterization of Isolated Compounds

*β*-*Eudesmol* (**1**). White needles. ^1^H-NMR (500 MHz, CDCl_3_) δ ppm: 4.74 (1H, s, H-11a), 4.47 (1H, s, H-11b), 2.33 (1H, m, H-3a), 2.01 (1H, m, H-3b), 1.79 (1H, m, H-5), 1.65 (2H, m, H-2), 1.55 (2H, m, H-1), 1.47 (2H, m, H-9), 1.40 (1H, m, H-7), 1.28 (2H, m, H-8), 1.23 (6H, s, H-13, H-14), 1.16 (2H, m, H-6), 0.72 (3H, s, H-15). ^13^C-NMR (125 MHz, CDCl_3_) δ ppm: 151.2 (C-4), 105.3 (C-11), 73.0 (C-12), 49.8 (C-7), 49.5 (C-5), 41.9 (C-9), 41.1 (C-1), 36.9 (C-3), 35.9 (C-10), 27.2 (C-14), 27.1 (C-13), 25.0 (C-6), 23.5 (C-2), 22.4 (C-8), 16.3 (C-15) [19].

*trans-3β-(1-Hydroxy-1-methylethyl)-8aβ-methyl-5-methylenedecalin-2-one* (**2**). White powder. ^1^H-NMR (500 MHz, CDCl_3_) δ ppm: 4.86 (1H, s, H-11a), 4.55 (1H, s, H-11b), 2.50 (1H, m, H-7), 2.41 (1H, m, H-3a), 2.37 (1H, m, H-5), 2.32 (1H, d, *J* = 10.0 Hz, H-9a), 2.20 (1H, d, *J* = 10.0 Hz, H-9b), 2.10 (1H, m, H-6a), 2.10 (1H, m, H-3b), 1.71 (1H, m, H-6b), 1.66 (1H, m, H-2a), 1.56 (1H, m, H-2b), 1.52 (2H, m, H-1), 1.29 (3H, s, H-14), 1.27 (3H, s, H-13), 0.72 (3H, s, H-15) [19]. ^13^C-NMR (125 MHz, CDCl_3_) δ ppm: 214.7 (C-8), 148.5 (C-4), 107.3 (C-11), 71.5 (C-12), 58.9 (C-7), 57.1 (C-9), 48.4 (C-5), 41.2 (C-1), 40.8 (C-10), 36.6 (C-3), 28.6 (C-13), 28.1 (C-6), 25.5 (C-14), 23.0 (C-2), 17.1 (C-15) [20].

*10-Methoxy-7-methyl-2H-benzo[g]chromen-2-one* (**3**). Colorless needles. HR-ESI-MS *m*/*z*: 241.0861 [M+H]^+^ (calcd. for C_15_H_13_O_3_, 241.0865). ^1^H-NMR (500 MHz, CDCl_3_) δ ppm: 8.33 (1H, d, *J* = 8.5 Hz, H-9), 8.21 (1H, d, *J* = 9.5 Hz, H-4), 7.53 (1H, s, H-6), 7.32 (1H, d, *J* = 8.5 Hz, H-8), 6.86 (1H, s, H-5), 6.47 (1H, d, *J* = 9.5 Hz, H-3), 4.03 (3H, s, 10-OCH_3_), 2.54 (3H, s, 7-CH_3_). ^13^C-NMR (125 MHz, CDCl_3_) δ ppm: 161.0 (C-2), 152.6 (C-10), 152.5 (C-11), 139.5 (C-7), 139.4 (C-4), 135.8 (C-13), 126.9 (C-8), 126.0 (C-6), 122.3 (C-9), 117.1 (C-14), 114.2 (C-3), 108.1 (C-12), 100.3 (C-5), 55.8 (10-OCH_3_), 21.9 (7-CH_3_).

*5,7-Dimethoxy-8-[(Z)-3'-methylbutan-1',3'-dienyl]coumarin* (**4**). Colorless needles. ^1^H-NMR (500 MHz, CDCl_3_) δ ppm: 8.01 (1H, d, *J* = 9.5 Hz, H-4), 6.42 (1H, d, *J* = 12.0 Hz, H-1'), 6.34 (1H, s, H-6), 6.17 (1H, d, *J* = 12.0 Hz, H-2'), 6.17 (1H, d, *J* = 9.5 Hz, H-3), 4.87 (2H, d, *J* = 12.0 Hz, H-4'), 3.98 (3H, s, 7-OCH_3_), 3.94 (3H, s, 5-OCH_3_), 1.63 (3H, s, H-5'). ^13^C-NMR (125 MHz, CDCl_3_) δ ppm: 161.4 (C-2), 160.4 (C-7), 156.2 (C-5), 152.9 (C-9), 142.6 (C-3'), 138.6 (C-4), 136.7 (C-1'), 117.1 (C-2'), 116.9 (C-4'), 111.1 (C-3), 108.1 (C-8), 103.6 (C-10), 90.1 (C-6), 56.0 (7-OCH_3_), 55.9 (5-OCH_3_), 20.7 (C-5') [21].

*7-Geranyloxy-6-methoxycoumarin* (**5**). Brown needles. ^1^H-NMR (500 MHz, CDCl_3_) δ ppm: 7.65 (1H, d, *J* = 9.5 Hz, H-4), 6.87 (1H, s, H-5), 6.84 (1H, s, H-8), 6.29 (1H, d, *J* = 9.5 Hz, H-3), 5.50 (1H, t, H-2'), 5.08 (1H, t, H-6'), 4.71 (2H, d, *J* = 6.0 Hz, H-1'), 3.93 (3H, s, 6-OCH_3_), 2.13 (2H, m, H-5'), 2.10 (2H, m, H-4'), 1.79 (3H, s, H-9'), 1.67 (3H, s, H-8'), 1.61 (3H, s, H-10'). ^13^C-NMR (125 MHz, CDCl_3_) δ ppm: 161.5 (C-2), 152.1 (C-7), 150.0 (C-9), 146.7 (C-6), 143.3 (C-4), 142.1 (C-3'), 131.9 (C-7'), 123.6 (C-6'), 118.5 (C-2'), 113.3 (C-3), 111.3 (C-10), 108.1 (C-5), 101.2 (C-8), 66.3 (C-1'), 56.4 (6-OCH_3_), 39.5 (C-4'), 26.2 (C-5'), 25.6 (C-8'), 17.7 (C-10'), 16.9 (C-9') [22].

*5,7-Dimethoxy-8-(3-methyl-2-oxobutyl)coumarin* (**6**). Colorless crystals. ^1^H-NMR (500 MHz, CDCl_3_) δ ppm: 8.01 (1H, d, *J* = 9.5 Hz, H-4), 6.34 (1H, s, H-6), 6.14 (1H, d, *J* = 9.5 Hz, H-3), 3.95 (3H, s, 5-OCH_3_), 3.93 (2H, s, H-1'), 3.89 (3H, s, 7-OCH_3_), 2.81 (1H, m, H-3'), 1.22 (3H, s, H-4'), 1.20 (3H, s, H-5'). ^13^C-NMR (125 MHz, CDCl_3_) δ ppm: 211.3 (C-2'), 161.3 (C-2), 161.2 (C-7), 156.2 (C-5), 153.9 (C-9), 138.9 (C-4), 110.9 (C-3), 104.1 (C-8), 103.8 (C-10), 90.2 (C-6), 56.0 (7-OCH_3_), 55.9 (5-OCH_3_), 40.7 (C-3'), 34.3 (C-1'), 18.5 (4'-CH_3_, 5'-CH_3_) [23].

*Murrangatin acetate* (**7**). Colorless needles. ^1^H-NMR (500 MHz, CDCl_3_) δ ppm: 7.64 (1H, d, *J* = 9.5 Hz, H-4), 7.42 (1H, d, *J* = 8.5 Hz, H-5), 6.90 (1H, d, *J* = 8.5 Hz, H-6), 6.28 (1H, d, *J* = 9.5 Hz, H-3), 5.75 (1H, d, *J* = 8.0 Hz, H-2'), 5.50 (1H, t, H-1'), 4.77 (2H, m, H-4'), 4.02 (3H, s, 7-OCH_3_), 3.60 (1H, d, *J* = 10.0 Hz, 1'-OH), 2.16 (3H, s, H-7'), 1.77 (3H, s, H-5'). ^13^C-NMR (125 MHz, CDCl_3_) δ ppm: 170.9 (C-6'), 160.2 (C-2), 160.0 (C-7), 152.7 (C-9), 143.7 (C-4), 140.8 (C-3'), 128.8 (C-5), 115.8 (C-8), 114.9 (C-4'), 113.5 (C-3), 113.1 (C-10), 107.8 (C-6), 79.5 (C-2'), 68.2 (C-1'), 56.3 (7-OCH_3_), 21.2 (C-7'), 18.6 (C-5') [24].

*Toddalenone* (**8**). Colorless needles. ^1^H-NMR (500 MHz, CDCl_3_) δ ppm: 8.00 (1H, d, *J* = 9.5 Hz, H-4), 7.95 (1H, d, *J* = 16.5 Hz, H-1'), 7.25 (1H, d, *J* = 16.5 Hz, H-2'), 6.35 (1H, s, H-6), 6.21 (1H, d, *J* = 9.5 Hz, H-3), 4.02 (3H, s, 7-OCH_3_), 4.01 (3H, s, 5-OCH_3_), 2.42 (3H, s, H-4'). ^13^C-NMR (125 MHz, CDCl_3_) δ ppm: 199.9 (C-3'), 163.1 (C-7), 160.4 (C-2), 158.3 (C-5), 155.0 (C-9), 138.5 (C-4), 131.8 (C-1'), 129.8 (C-2'), 111.4 (C-3), 104.7 (C-8), 103.8 (C-10), 90.2 (C-6), 56.2 (7-OCH_3_), 56.1 (5-OCH_3_), 27.6 (4'-CH_3_) [25].

### 3.5. Cytotoxicity Assay

The cytotoxicity of compounds **1**–**8** was measured by the standard CCK-8 method [26]. Human lung adenocarcinoma (A549), human hepatocellular carcinoma cells (SMMC-7721), human bladder tumor cells (EJ), human cervical carcinoma cells (Hela), and human B-lineage acute lymphoblastic leukemia 1 cells (BALL-1) were purchased from the Chinese Academy of Medical Sciences (Beijing, China). Doxorubicin (DOX, adriamycin, Actavis Italy S.p.A., Beijing, China) was the positive control. All cell lines were cultured in RPMI 1640 (Sigma, St. Louis, MO, USA) medium supplemented with 10% fetal bovine serum (GIBCO Inc., Grand Island, NY, USA), 100 IU/mL penicillin (Flow Lab, Beijing, China) and 100 μg/mL streptomycin (Flow Lab) at 37 °C, 5% CO_2_ and 90% humidity. The cell suspension was dispensed into a 96-well plates at 100 μL per well (adherent cells were 6 × 10^3^ per well, suspension cells were 5 × 10^4^ per well). After 4–6 h preincubation in the incubator (Forma Series ΙΙ Water Jacket, Waltham, MA, USA) to allow cellular attachment, various concentrations of test solution were added and cells were incubated for 48 h. At the end of the incubation, CCK-8 reagent (Dojindo, Kumamoto, Japan, 10 μL) was added into each well followed by further incubation for 2 h. The optical density (OD) was recorded at 450 nm using a microplate reader (Multiskan GO, Thermo Scientific, Waltham, MA, USA). Each determination represented the average mean of six replicates. The 50% inhibitory concentration (IC_50_) values were calculated by the line equation of the dose-dependent curves.

## 4. Conclusions

A new compound and seven known compounds were isolated from the *M. tetramera* for the first time. They were two sesquiterpenes (compounds **1** and **2**) and six coumarins (compounds **3**–**8**). All the compounds were tested for their *in vitro* cytotoxic activities against the HeLa, K562, A549, H1299 and SMMC-7721 tumor cell lines. It was found that compounds with similar structures displayed various degrees of cytotoxicity. Compound **1** showed stronger cytotoxic activity against the five cell lines (A549, SMMC-7721, EJ, HeLa and BALL-1) compared to compound **2**. Compound **5** showed potent cytotoxicity against three cell lines (A549, SMMC-7721 and BALL-1). Compound **4** showed promising cytotoxicity against three cell lines (A549, EJ and BALL-1). Compound **3** only showed cytotoxicity against the BALL-1 cell line. None of the tested cell lines were susceptible towards compounds **6**–**8**. This phenomenon might be related with the different substitution patterns in their chemical structures. Compounds having long alkyl-substituents exhibited significant potent cytotoxic activity against the human cancer cell lines, whereas compounds which had carbonyl groups on the alkyl substituents showed weak cytotoxicity. All these active compounds might be promising lead compounds for anti-cancer agents. However, further study is needed to unravel the mechanisms of their cytotoxic activity.
